# Efficient Congo Red Removal Using Porous Cellulose/Gelatin/Sepiolite Gel Beads: Assembly, Characterization, and Adsorption Mechanism

**DOI:** 10.3390/polym13223890

**Published:** 2021-11-10

**Authors:** Chenlu Jiao, Die Liu, Nana Wei, Jiannan Gao, Fan Fu, Tao Liu, Jian Wang

**Affiliations:** College of Light-Textile Engineering and Art, Anhui Agricultural University, Hefei 230036, China; shangyali2021@163.com (D.L.); zhangxiaoli201909@163.com (N.W.); mazheng2021@126.com (J.G.); chenyysuda@126.com (F.F.); wangjiaqing20@126.com (T.L.)

**Keywords:** microcrystalline cellulose, gelatin, sepiolite, beads, dye, adsorption

## Abstract

Porous sustainable cellulose/gelatin/sepiolite gel beads were fabricated via an efficient ‘hydrophilic assembly–floating droplet’ two-step method to remove Congo red (CR) from wastewater. The beads comprised microcrystalline cellulose and gelatin, forming a dual network framework, and sepiolite, which acted as a functional component to reinforce the network. The as-prepared gel beads were characterized using FTIR, SEM, XRD, and TGA, with the results indicating a highly porous structure that was also thermally stable. A batch adsorption experiment for CR was performed and evaluated as a function of pH, sepiolite addition, contact time, temperature, and initial concentration. The kinetics and isotherm data obtained were in agreement with the pseudo-second-order kinetic model and the Langmuir isotherm, with a maximum monolayer capacity of 279.3 mg·g^−1^ for CR at 303 K. Moreover, thermodynamic analysis demonstrated the spontaneous and endothermic nature of the dye uptake. Importantly, even when subjected to five regeneration cycles, the gel beads retained 87% of their original adsorption value, suggesting their suitability as an efficient and reusable material for dye wastewater treatments.

## 1. Introduction

Nowadays, wastewaters from dye-using industries, such as clothing, leather, synthesis, and electroplating, pose a major challenge to global society [1]. Of the various types of environmental harm caused by these industries, the aquatic environmental contamination by azo dyes is considered the most serious. Congo red (CR), a popular anionic azo dye, is intrinsically harmful to living organisms [2,3]. Even the presence of very low concentrations of CR in wastewater imparts a color, which blocks light and inhibits the photosynthetic efficiency of aquatic life [4]. Moreover, these effluents are highly toxic and non-biodegradable to humans, fauna, and flora, with some variants being carcinogenic and mutagenic [5]. Considering their complex aromatic structure, thermal stability, and stable chemistry, the treatment of CR-containing effluents before being discharged into any natural resource is critical [6].

Considerable effort has been put into developing techniques to remove CR from effluents, including photolysis [7], coagulation/flocculation [8], adsorption [9], membrane separation [10], and electrochemical processes [11]. Among these methods, adsorption is recognized as a practical solution, owing to its low cost, ease of operation, lack of secondary pollution, and potential for regeneration [12]. A well-known adsorbent for CR removal is activated carbon, which is effective for a range of pollutants, including dyes and pigments. However, the high cost and difficulty associated with its regeneration has limited extensive use [13], and, predictably, focus has shifted to the development of renewable adsorbents based on natural and low-cost materials.

In this regard, natural biomass materials have garnered widespread attention in recent years, due to their abundant sources, eco-friendliness, high uptake capacity, possible regeneration, and universality [14]. Cellulose, a typical bio-renewable and biodegradable natural polymer, consists of a liner chain of thousands of *β*-(1→4)-linked *d*-glucose units. Since the successful preparation of cellulose aerogels by Tan et al. in 2001, the field has progressed rapidly with third-generation cellulose aerogel materials today being widely used in adsorption, energy storage, thermal resistance, electromagnetic interference shielding, and biomedicine [15,16]. Even so, the large-scale utilization of pure cellulose aerogels for dye removal from effluents is still limited, on account of the unsatisfactory structural properties associated with them, such as fragile collapse, weak mechanical strength, and limited adsorption. To optimize the structure and performance of cellulose aerogels, three strategies are commonly adopted: (I) mixing two or more polymers to build multiple networks [17,18]; (II) assembling high-strength materials at mesoscopic scale [19]; (III) doping organic or inorganic components to enhance the adsorption capacity [20,21].

Gelatin (GEL) is a protein biopolymer derived from collagenous animal tissues by hydrolysis and has been explored for alternative wound dressings, drug carriers, adsorbents, and tissue scaffolds, owing to its low cost, water solubility, biocompatibility, and biodegradability [19,22]. The abundant functional groups, such as hydroxyl, carboxyl, and amino, on the backbone of GEL make its gelation and functionalization easy, leading to its use as a component to build 3D net structures [23,24]. Herein, in view of the good compatibility of cellulose and GEL, a cross-linked dual network is built using the electrostatic interaction and hydrogen bond [24,25]. On the one hand, the prepared dual network strengthens the mechanical strength and structural stability of the gel, providing a good foundation for recycling, while on the other, the dual network facilitates the capture of dye molecules, to further improve the adsorption capacity.

Recently, hybrid materials composed of organic polymers and inorganic molecules have attracted widespread attention, owing to the synergetic effects of combining the properties of organic polymers (flexibility, toughness, and processability) with those of inorganic molecules (heat resistance, rigidity, and favorable mechanical strength) [26,27]. Sepiolite (SEP) is a natural hydrated magnesium silicate clay with the unit cell formula Si_12_Mg_8_O_30_(OH)_4_(H_2_O)_4_·8H_2_O [28]. SEP has tubular channels with a cross-sectional area of 0.36 nm × 10.6 nm and a theoretical specific surface area of up to 900 m^2^·g^−1^. This is the largest of natural inorganic minerals, comparable even to activated carbon, which is a powerful adsorbent of dyes [20]. Furthermore, the presence of numerous silanol groups on the surface of SEP facilitates strong interaction with cellulose and GEL chains, contributing improved properties to hybrid materials.

To the best of our knowledge, a detailed study on the synthesis of cellulose/gelatin/sepiolite gel beads has not yet been reported. Herein, a type of porous sustainable microcrystalline cellulose/gelatin/sepiolite (MGS) gel beads were fabricated via a simple and efficient ‘hydrophilic assembly–floating droplet’ two-step method, in which microcrystalline cellulose and gelatin were selected as dual network frameworks and SEP acted as a functional component to optimize the network. The assembly mechanism, morphology, structure, and thermal stability of the hybrid beads were investigated. In particular, the CR removal performance of the hybrid beads was studied as a function of SEP addition, pH, contact time, temperature, and initial CR concentrations. Furthermore, the kinetics, isotherms, and thermodynamics of the CR adsorption process were also thoroughly investigated.

## 2. Materials and Methods

### 2.1. Materials

Microcrystalline cellulose (MCC, 20–80 μm) and gelatin (GEL) were purchased from Aladdin Industrial Co., Ltd. (Shanghai, China). Natural sepiolite (SEP) with 99% pure mineral content was purchased from Sigma-Aldrich Co., Ltd. (Shanghai, China) and was filtered through a 200 mesh standard sieve followed by treatment with 6 mol·L^−1^ HCl for 6 h before blending. Sodium hydroxide (NaOH), urea, hydrochloric acid (HCl), and calcium chloride (CaCl_2_) were obtained from Sinopharm Chemical Reagent Co., Ltd. (Shanghai, China). Congo red (CR) was purchased from Shanghai Titan Technology Co., LTD. (Shanghai, China).

### 2.2. Synthesis of MGS Gel Beads

**Figure 1:**
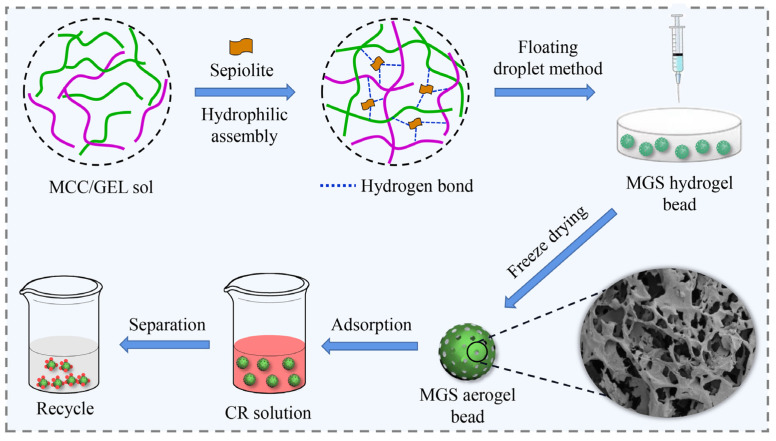
Schematic diagram for the fabrication of MGS gel beads and adsorption of CR dye.

First, 100 g SEP was added to 1 L 15% HCl solution, with stirring for 24 h, then washed with distilled water until neutral pH and dried for usage [29]. Then, an aqueous solution of 7 wt% NaOH/12 wt% urea was prepared and precooled to −23 °C. Next, 3 g MCC and 1 g GEL were immediately added to the 100 mL NaOH/urea solution under vigorous stirring for 10 min to obtain a 4 wt% homogeneous MCC/GEL hybrid sol. Then, 1.5 g pretreated SEP was added into the hybrid sol and stirred for 2 h. The resulting suspension was added dropwise using a 5 mL syringe at a dropping rate of 2 mL·min^−1^ into the HCl solution containing 5 wt% CaCl_2_ for 12 h to solidify. The well-formed MCC/GEL/SEP (MGS-1.5, according to the mass of SEP) hydrogel beads were filtered and washed in deionized water to remove residual chemicals, and MGS-1.5 gel beads were obtained after freeze-drying. A schematic diagram for the generalized fabrication process is shown in Figure 1. For comparison, MGS gel beads with 0 g, 0.5 g, 1.0 g, and 2.0 g SEP were synthesized using the same process.

### 2.3. Characterization of MGS Gel Beads

The surface morphology of MGS gel beads was studied using field emission scanning electron microscopy (FE-SEM, Hitachi S-4800, Tokyo, Japan). The crystalline and chemical structures were recorded using X-ray diffractometry (XRD, Bruker D8, Bremen, Germany) and Fourier transform infrared spectroscopy (FT-IR, Thermo Nicolet 5700, Carlsbad, CA, USA). A thermal analyzer (TG, TA-SDTQ600, Waltham, MA, USA) was used to survey the weight loss of the MGS gel beads from ambient conditions to 600 °C, at a heating rate of 10 °C·min^−1^ in N_2_.

### 2.4. Density and Porosity Measurement

The density of gel beads was calculated from the weight and volume of the specimen, while the porosity was determined using a liquid displacement method, with absolute ethanol as a solvent [30]. The weighed gel beads were dipped into a petri dish containing absolute ethanol, until they approached saturation, following which they were taken out and the residual ethanol weight was again obtained. The porosity of the beads was then calculated according to the following equation:

(1) $$\mathrm{Porosity}=(\frac{W_{2}-W_{1}-W_{3}}{W_{2}-W_{3}})\times 100\%$$

where *W*_1_ is the weight of the beads, *W*_2_ represents the sum of the weights of the immersed beads and ethanol, and *W*_3_ denotes the weight of the residual ethanol after the beads were removed.

### 2.5. Adsorption and Desorption of CR Dye

The batch adsorption experiment of CR from aqueous solutions was carried out as follows: 0.1 g MGS gel beads was soaked in 100 mL CR solution, with various concentrations, and stirred for different durations. The initial pH of the CR solutions was adjusted using dilute HCl or NaOH solutions. UV-visible spectroscopy (UV-vis, TU-1810, Purkinje General, Beijing, China) was employed to analyze the concentrations of CR at 497 nm, before and after adsorption. The adsorption capacity was calculated according to the following equation:

(2) $$Q_{t}=\frac{(C_{o}-C_{t})V}{m}$$

where *Q*_t_ (mg·g^−1^) is the adsorbed capacity at time t (min), *C*_o_ (mg·L^−1^) and *C*_t_ (mg·L^−1^) are the CR concentrations at initial and given time t (min), respectively. *V* corresponds to the volume of CR solution (L) and *m* denotes the weight of MGS gel beads (g). The adsorption data at different time intervals were used to fit the kinetic curves of CR adsorption, and the isotherm parameters were obtained by comparing the adsorption quantities under different initial concentrations.

For regeneration, the MGS gel beads were desorbed after adsorption via 0.05 mol·L^−1^ NaOH eluent, with stirring at 303 K for 4 h. After the complete elution, the MGS gel beads were washed and dried to obtain the regenerated beads. To evaluate the reusability of the regenerated MGS gel beads, five such sequential adsorption–desorption cycles were carried out.

## 3. Results and Discussion

### 3.1. Fabrication Principle and Strategy of MGS Gel Beads

As displayed in Figure 1, an efficient ‘hydrophilic assembly–floating droplet’ two-step method and possible assembly mechanism are proposed. First, MCC and GEL chains worked as framework materials to assemble a dual network. When embedding SEP with abundant silicon hydroxyl groups, an effective ‘hydrophilic assembly’ occurs, driven by the hydrophilic groups (silicon hydroxyl groups) of SEP sheets and active sites (oxygen-containing groups) of the dual network structure via an electrostatic interaction and hydrogen bonding, leading to an interpenetrating network structure [19,31]. Then, a ‘floating droplet’ technology was employed to obtain homogeneous porous MGS beads via sol–gel conversion induced by a CaCl_2_/HCl coagulating bath. Hence, in the assembly process, SEP sheets acted like a ‘crosslinker’ to connect the dual network and construct uniform and robust beads with good resistance to deformation. Furthermore, SEP sheets with abundant silicon hydroxyl groups are expected to improve the specific surface area and adsorption capacity of MGS beads.

### 3.2. Chemical Analysis of MGS Gel Beads

**Figure 2:**
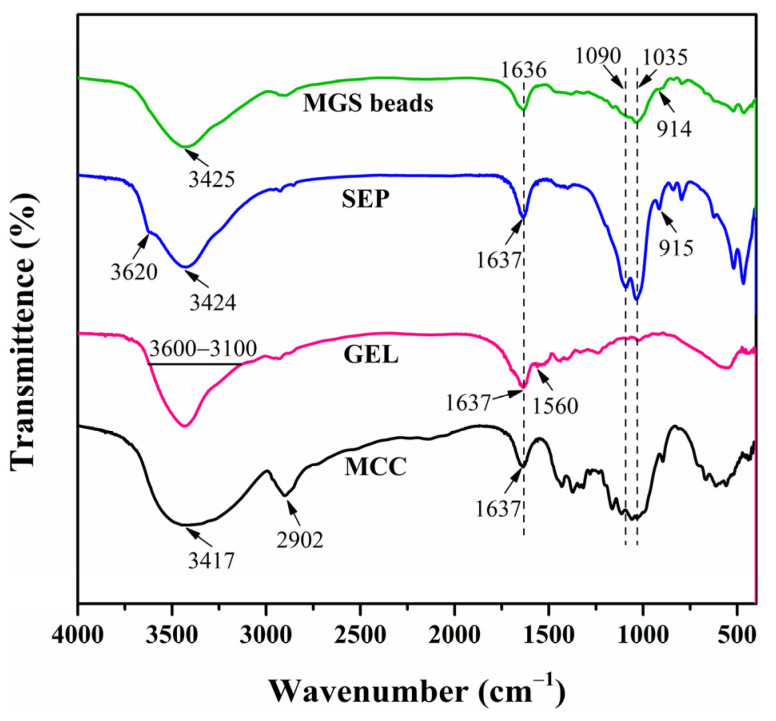
FTIR spectra of MCC, GEL, SEP, and MGS-1.5 beads.

FTIR spectroscopy was performed on the MCC, GEL, SEP, and MGS beads to understand their chemical structure, and the respective spectra are displayed in Figure 2. As can be seen in Figure 2, the bands of MCC at 3417 and 1637 cm^−1^ represent O–H stretching and bending vibrations, respectively [32]. The peak at 2902 cm^−1^ is assigned to C–H stretching vibration [33]. In the spectrum of GEL, a broad band in the range of 3600–3100 cm^−1^ was attributed to N–H and O–H stretching vibrations [34,35], while those at 1637 and 1560 cm^−1^ are designated as C=O stretching (amide I) and N–H bending vibrations (amide II) [36]. The characteristic peak at 3620 cm^−1^ for SEP corresponds to the stretching vibration of the Mg–OH group in the octahedral layers, and the absorbance at 3424 and 1637 cm^−1^ were assigned to the vibrations of zeolitic water or structurally bound water. Next, the bands at 915 and 1090 cm^−1^ were attributed to the asymmetrical vibration of Si–O, while the band at 1035 cm^−1^ corresponds to Si–O–Si plane vibration [28,37]. As for the MGS beads, peaks at 1090, 1035, and 916 cm^−1^ appeared in the spectrum of MGS, indicating the involvement of SEP in the hybrid gel beads. Moreover, the bands assigned to O–H stretching and bending vibrations shifted to 3425 and 1636 cm^−1^, respectively. The stretching vibration peak of C–H at 2800~3000 cm^−1^ decreased, indicating that C–H may participate in the cross-linking reaction [18]. The Mg–OH stretching vibration at 3620 cm^−1^ assigned to the free silanol groups located on the external surface of SEP disappeared in the spectrum of the MGS bead. A similar phenomenon was observed in related hybrid systems and correlated to the presence of hydrogen bonding interactions between the silanol groups of SEP and hydrophilic matrices [38,39,40]. Such favorable interactions are responsible for the good dispersion of SEP, MCC, and GEL molecules, generating porous hybrid beads with an improved performance and adsorption capacity.

### 3.3. Structural Characterization of MGS Gel Beads

**Figure 3:**
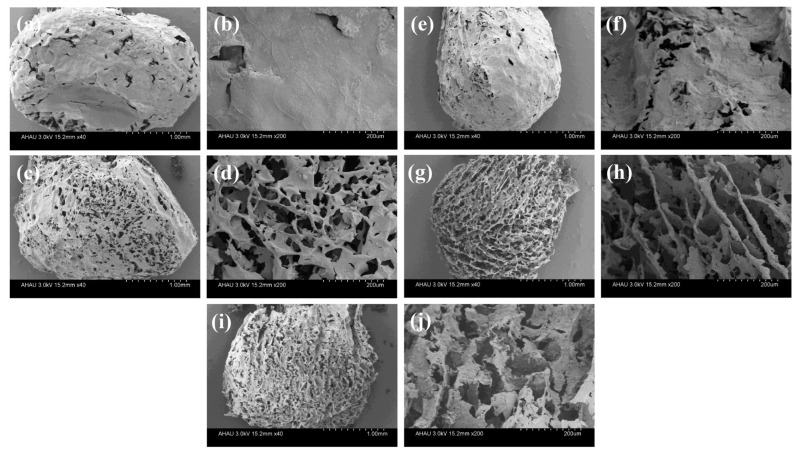
FE-SEM micrographs of different MGS gel beads assemblied with various additions of SEP: (**a**,**b**) MGS-0, (**c**,**d**) MGS-0.5, (**e**,**f**) MGS-1.0, (**g**,**h**) MGS-1.5, (**i**,**j**) MGS-2.0.

To gain an insight into the structural characteristics of MGS beads, the morphology of MGS beads with various SEP additions was studied using field emission scanning electron microscopy (FE-SEM). As displayed in Figure 3, all beads showed a diameter of ca. 3 mm, and the MGS-0 bead (Figure 3a,b) exhibited few pores, with a relatively smooth surface. With the addition of SEP (Figure 3c–h), a highly-porous structure was observed, which is crucial to facilitating dye adsorption. Specifically, the incorporation of SEP into the dual network provoked a change in the interconnected network, reflecting a layered porous network, as confirmed by Figure 3c–h. Furthermore, the surface of the layered structure became progressively rougher, which is the typical morphology of SEP sheets and led to an improved specific surface area of the MGS beads. When the SEP content increased further to 2.0 g (Figure 3i,j), the pore size became smaller, and the layered structure tended to accumulate and collapse, which was due to the fragmentation of the bead structure caused by the impaired load transfer of excessive SEP through the dual network.

**Figure 4:**
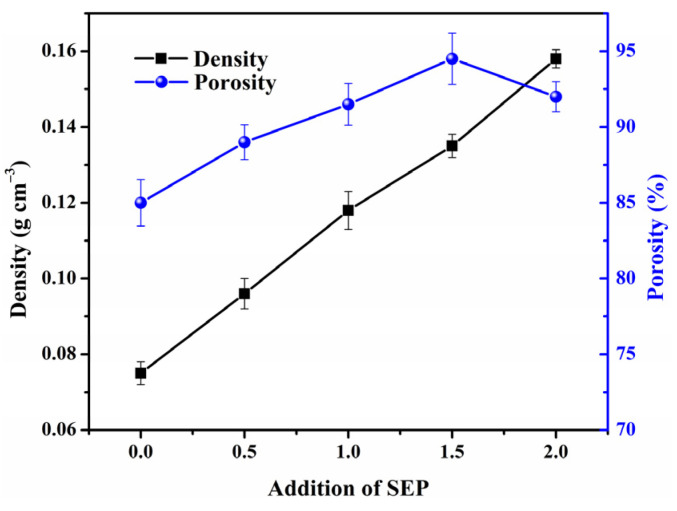
Density and porosity of MGS beads as a function of the SEP addition.

The variation of porosity and density with the addition of SEP is shown in Figure 4, and the MGS beads were found to be lightweight and full of pores. The density of beads varied from 0.075 to 0.158 g·cm^−3^, and showed an almost linear increase with respect to the addition of SEP. Unexpectedly, the porosity was enhanced with increasing SEP content initially, followed by a decrease, with the maximum porosity occurring at a SEP content of 1.5 g SEP. This variation pattern is consistent with the analysis of SEM photos, which further demonstrates that changes of the assembled structure of MGS beads can be induced by SEP. Taking into consideration the nature of the density and porosity changes, 1.5 g SEP was chosen as the most suitable for this study.

### 3.4. XRD Analysis of MGS Gel Beads

**Figure 5:**
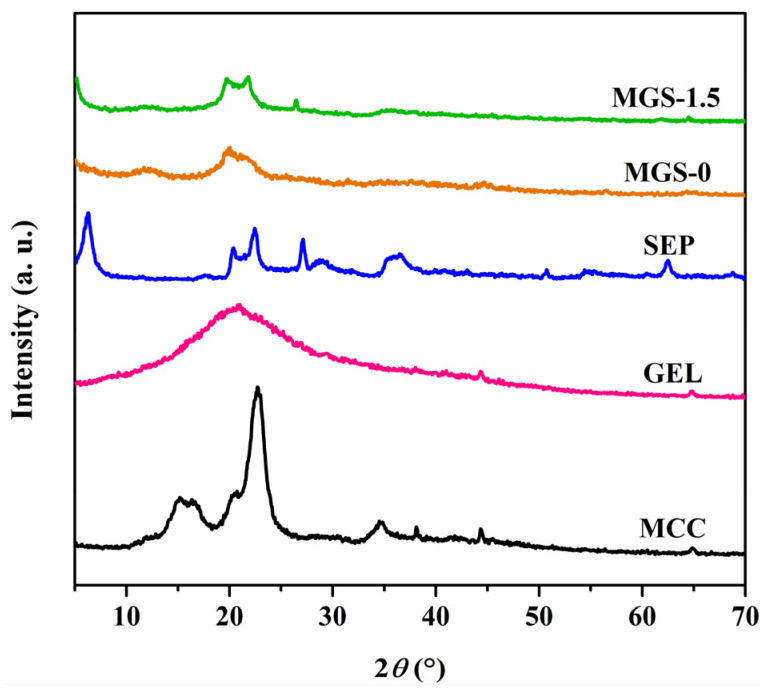
XRD patterns of MCC, GEL, SEP, MGS-0, and MGS-1.5 beads.

XRD patterns of the MCC, GEL, SEP, and MGS-1.5 beads were investigated and are shown in Figure 5. The diffraction peaks at 2θ of 15.20°, 16.35°, 22.70°, and 34.85° can be attributed to 101, 10$\bar{1}$, 002, and 040 lattice planes of MCC and are characteristic of the cellulose I crystal structure [41]. The XRD pattern of GEL shows a broad peak at 2θ ~ 20.20°, indicating the slightly amorphous nature of GEL [42]. As for SEP, the peaks at 2θ of 6.30° and 27.15° correspond to 110 and 080 reflections of silicate, respectively [43]. Compared with the MGS-0 beads, some characteristic SEP peaks appeared in the MGS-1.5 bead, indicating the homogenous dispersion of SEP in the bead matrix, due to its sheets being intercalated into the dual network [20,44]. Unexpectedly, no obvious MCC diffraction peaks appeared in the MGS-1.5 beads. This can be attributed to changes in the crystal structure of MCC, to resemble cellulose II, brought about by the dissolution of the alkali/urea system, as confirmed by peaks at 2θ of 12.10°, 20.05°, and 21.05° in the MGS-0 beads [45].

### 3.5. Thermal Property of MGS Gel Beads

**Figure 6:**
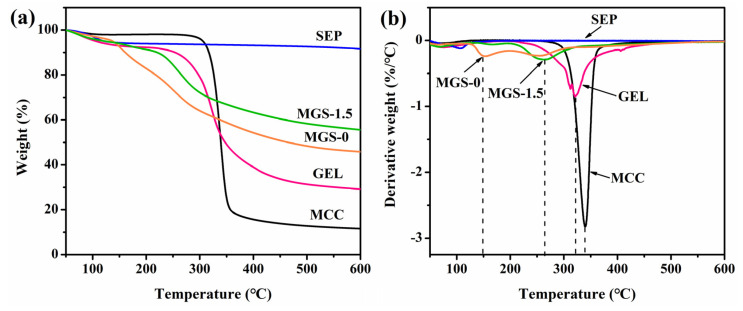
TGA (**a**) and DTG (**b**) curves obtained for MCC, GEL, SEP, MGS-0, and MGS-1.5 beads.

As shown in Figure 6, the thermal stability and the extent of degradation of MGS beads and components was investigated using TGA and DTG analyses. As for SEP, a total mass loss of approximately 8.4% over the temperature range of 30–600 °C was observed, as shown in Figure 6a. The weight residual rate of MGS-1.5 beads (55.6%) was higher than that of MCC (11.6%), GEL (29.2%), and MGS-0 beads (45.8%), which can be attributed to the addition of thermally stable SEP (91.6%). Moreover, the increased uniform molecular dispersion and interfacial interactions between polar polymer groups and silicate layers of SEP potentially enhanced the thermal stability of the MGS-1.5 bead. Regarding the derivative weight loss curves (DTG in Figure 6b), a weak mass loss peak was observed at ~100 °C for SEP, confirming the presence of zeolitic or structurally bound water on the SEP surface. The decomposition of MGS-0 was found to occur at 147 °C and 253 °C, and was associated with the elimination of the physically adsorbed water molecules in the first step. In the second stage, hydroxyl groups within the MGS-0 beads underwent dehydration and part of the glycoside bonds in the beads broke, resulting in rearrangement of the chemical bonds. In contrast with MGS-0, the decomposition temperature of the MGS-1.5 beads was 262 °C, significantly higher than the MGS-0 beads. Thus, as expected, the hybrid beads showed a higher heat resistance than the MGS-0 beads, due to the confinement and thermal insulation effect of inorganic molecules in SEP [46].

### 3.6. Effect of pH and SEP Content on CR Adsorption

**Figure 7:**
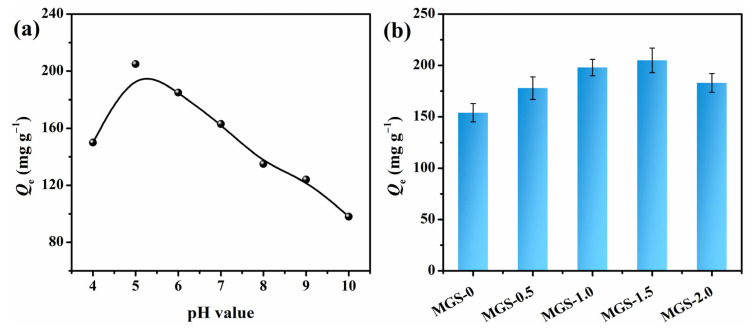
(**a**) Effect of pH value on the adsorption of CR on MGS-1.5 beads, and (**b**) adsorption capacities of MGS beads with various SEP additions for CR.

The pH value of the solution played a considerable role in the sorption study, as the surface charge of both the dye molecules and MGS beads varied significantly based on the pH. As depicted in Figure 7a, it was observed that the equalized adsorption amount (*Q*_e_) of CR was higher at a pH of 5.0 but less in alkaline and concentrated acidic environments. In detail, CR has a p*K*_a_ value of 4.5–5.5, and is positively charged, due to the protonation of nitrogen atoms and sulfonate groups, when the pH is less than 5.0, resulting in electrostatic repulsion between the protonated MGS beads and positive CR molecules [47]. In alkaline conditions, an electrostatic repulsion still existed between the deprotonated MGS beads and anionic CR molecules, generating a lower adsorption capacity. This phenomenon demonstrated that the adsorption process is significantly influenced by pH, and it was decided to set the pH value at 5.0 for subsequent studies.

As shown in Figure 7b, the *Q*_e_ values of MGS beads with various SEP additions for CR were investigated. After loading SEP, the *Q*_e_ gradually increased from 154 mg·g^−1^ to 205 mg·g^−1^ for MGS-0 and MGS-1.5 beads, respectively. This improved adsorption capacity of the MGS beads can be attributed to the increased surface area, enhanced porosity, and abundant functional groups. For MGS-2.0 beads, the *Q*_e_ decreased to 183 mg·g^−1^, due to the increased density and decreased porosity, which is consistent with the density, porosity, and SEM analyses.

### 3.7. Adsorption Kinetics

**Figure 8:**
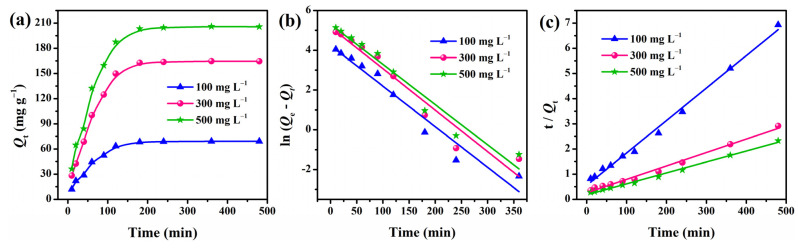
(**a**) Adsorption behaviors of MGS-1.5 beads for CR at various initial concentrations. (**b**) Pseudo-first-order and (**c**) pseudo-second-order kinetic models of MGS-1.5 beads.

The adsorption behavior of MGS-1.5 beads for CR at various initial concentrations (100–500 mg·L^−1^) was investigated at 303 K, and the results are displayed in Figure 8a and Table 1. It was evident that the amount of adsorption increased when raising the initial CR concentration from 100 to 500 mg·L^−1^. This was attributed to the significant driving forces for adsorption generated by the pressure gradient at higher concentrations. In addition, the adsorption process was fast during the first 120 min, and then leveled off gradually at about 180 min for all concentrations. To further investigate the adsorption mechanism and control the residual time of the adsorption process, the experimental data were analyzed using Lagergren’s pseudo-first-order and second-order models [48,49], which are given by Equations (3) and (4):

(3) $$\ln(Q_{1e}-Q_{t})=\ln Q_{1e}-k_{1}t$$

(4) $$\frac{t}{Q_{t}}=\frac{t}{Q_{2e}}+\frac{1}{k_{2}{Q_{2e}}^{2}}$$

where *Q*_1e_ (mg·g^−1^) and *Q*_2e_ (mg·g^−1^) are the adsorption capacities calculated from pseudo-first-order and pseudo-second-order kinetic models, respectively. *k*_1_ (min^−1^) and *k*_2_ (g·(mg min)^−1^) refer to the rate constants estimated from the fitting equations.

The fitting plots according to the pseudo-first-order and pseudo-second-order models are shown in Figure 8b,c, and all the corresponding kinetic parameters are summarized in Table 1. The correlation coefficients (*R*^2^ > 0.99) estimated from the pseudo-second-order kinetics for all concentrations were higher than those obtained from the pseudo-first-order kinetics. This can be further supported by the good agreement between the *Q*_2e_ values estimated from the pseudo-second-order models and the experimental *Q*_exp_ values. Thus, it was clear that the pseudo-second-order model describes the adsorption process accurately.

### 3.8. Adsorption Isotherm

**Figure 9:**
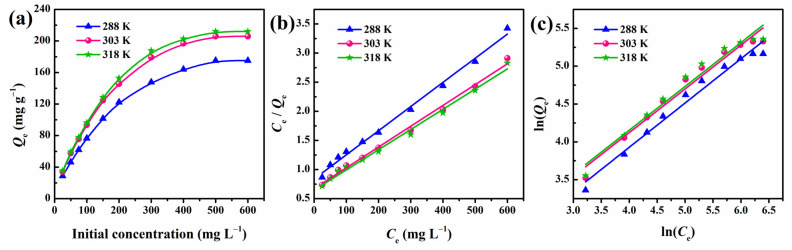
(**a**) Effect of initial concentration on absorption capacity of MGS-1.5 beads for CR. (**b**) Langmuir and (**c**) Freundlich adsorption isotherm plots of MGS-1.5 beads.

As shown in Figure 9a, the influence of initial concentration on the adsorption capacity was evaluated at different temperatures. It is clear that the adsorption capacity increased gradually as the CR concentration increased from 25 to 500 mg·L^−1^, before reaching saturation, which can be attributed to the increasing driving force generated by the concentration gradient, consistent with the results in Figure 8a. Furthermore, the adsorption rate increased with temperature, and the adsorption capacity also showed a small degree of enhancement.

To describe the interactive behavior between the CR and MGS beads and understand the CR molecular distribution in the liquid/solid phase at equilibrium, two classical adsorption isotherm equations, namely the Langmuir and Freundlich isotherms, were applied to build reliable predictive models. Assuming that the adsorption occurs via CR monolayer coverage onto a homogeneous adsorbent, with identical surface sites, the Langmuir isotherm can be expressed as in Equation (5) [50]:

(5) $$\frac{C_{e}}{Q_{e}}=\frac{C_{e}}{Q_{\max}}+\frac{1}{k_{L}Q_{\max}}$$

where *C*_e_ (mg·L^−1^) is the equilibrium concentration, *Q*_max_ (mg·g^−1^) stands for the maximum monolayer adsorption capacity per unit mass of MGS beads, and *k*_L_ (L·mg^−1^) is the Langmuir constant related to the energy of the adsorption process. Moreover, another essential parameter *R*_L_, which is a dimensionless constant, is defined by Equation (6):

(6) $$R_{L}=\frac{1}{1+k_{L}C_{o}}$$

where *R*_L_ indicates whether the Langmuir model is unfavorable (*R*_L_ > 1), favorable (0 < *R*_L_ < 1), linear (*R*_L_ = 1), or irreversible (*R*_L_ = 0).

The second isotherm, that of Freundlich, is described by assuming a heterogeneous surface with multilayer adsorption [51]. Its linear form is expressed as follows:

(7) $$\ln Q_{e}=\ln k_{F}+\frac{1}{n}\ln C_{e}$$

where *k*_F_ (L·mg^−1^) and *n* represent the Freundlich constant and the heterogeneity factor, which reflect the adsorption capacity and adsorption intensity, respectively.

The linearized curves and calculated parameters of the Langmuir and Freundlich isotherm models are presented in Figure 9 and Table 2, respectively. It is clear that all the regression coefficients from the Langmuir model (*R*^2^ > 0.99) at different temperatures fitted better than those of the Freundlich model (*R*^2^ > 0.96). In addition, favorable *R*_L_ (0.2352–0.8889) and reasonable Langmuir constant (*k*_L_ > 0) also reflect that the Langmuir model fit the experimental data well. This result indicates that CR adsorption occurs at a homogeneous MGS surface with identical binding sites, with the maximum monolayer adsorption reaching 279.3 mg·g^−1^ at 303 K.

### 3.9. Adsorption Thermodynamics

**Figure 10:**
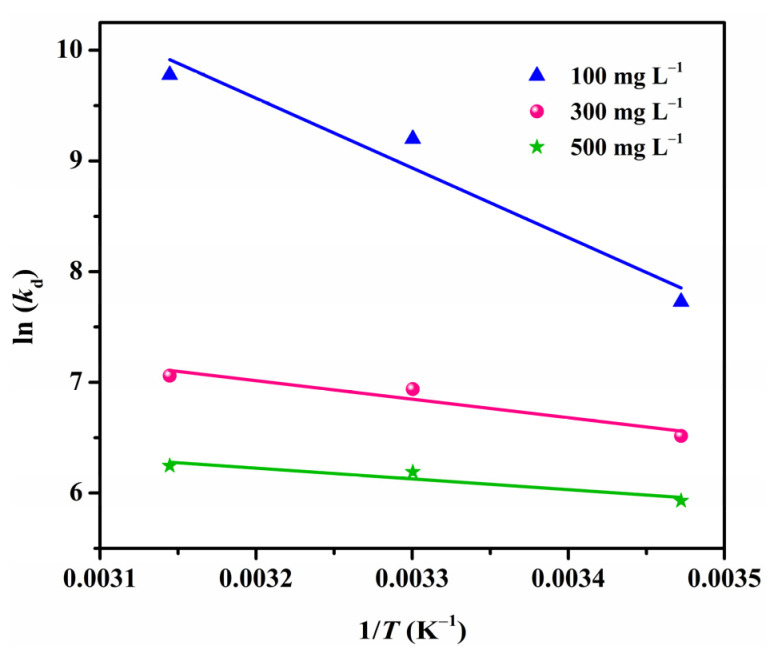
Van’t Hoff plot for adsorption of CR onto MGS-1.5 beads.

In order to gain in-depth information regarding the inherent energetic changes associated with the adsorption and the feasibility of the process, the adsorption thermodynamics were investigated assuming an isolated system, wherein the entropy change (Δ*S*°) is the only driving force (Figure 10) [52]. The thermodynamic parameters, such as enthalpy change (Δ*H*°), entropy change (Δ*S*°), and Gibbs free energy change (Δ*G*°), were determined using the Van’t Hoff equations as follows [53]:

(8) $$\ln(k_{d})=\frac{\Delta S^{{}^{\circ}}}{R}-\frac{\Delta H^{{}^{\circ}}}{RT}$$

where *R* and *T* stand for universal gas constant (8.314 J·(mol·K)^−1^) and solution temperature (K), respectively. Δ*H*° and Δ*S*° are determined from the slope and intercept of the ln(*k*_d_) plot vs. 1/*T*, in which *k*_d_ (L·mol^−1^) is the equilibrium constant obtained using Equation (9):

(9) $$k_{d}=\frac{Q_{e}}{C_{e}}$$

where *Q*_e_ (mg·L^−1^) and *C*_e_ (mg·g^−1^) are the adsorption amount and adsorbate concentration at equilibrium, respectively. Finally, Δ*G*° is calculated using the following relation:

(10) $$\Delta G^{{}^{\circ}}=\Delta H^{{}^{\circ}}-T\Delta S^{{}^{\circ}}$$

All the calculated values of Δ*H*°, Δ*S*°, and Δ*G*° are presented in Table 3. The positive Δ*H*° values reveal that the adsorption process was endothermic in nature, which can be supported by the fact that the adsorption of CR onto MGS beads increased when raising the temperature (Figure 9a). The values of Δ*S*° were found to be positive as well, suggesting an increase in randomness at the adsorbent/adsorbate interface during adsorption. This may be due to the fact that more translational entropy is gained by displacing adsorbed water with CR molecules than in losing it, thus causing increased randomness in the system [2]. The spontaneity of the adsorption process is confirmed by the negative Δ*G*° values in the studied ranges of temperature and concentration. Moreover, the increasing absolute values of Δ*G*° with increasing temperature reflect a more feasible adsorption process at high temperatures for CR, which is consistent with the experimental results and positive Δ*H*° values.

### 3.10. Stability and Reusability Studies

**Figure 11:**
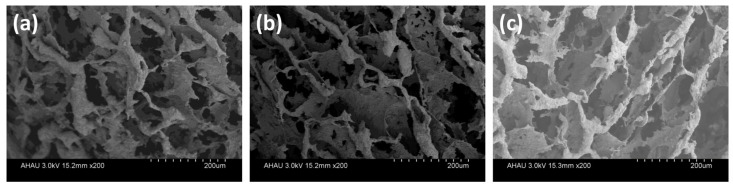
Micrographs of MGS-1.5 beads after adsorption in 100 mL 500 mg/L CR solution for 180 min with various conditions: (**a**) pH = 4 adjusted by HCl, (**b**) pH = 10 adjusted by NaOH, (**c**) 10 mL H_2_O_2_ solution.

**Figure 12:**
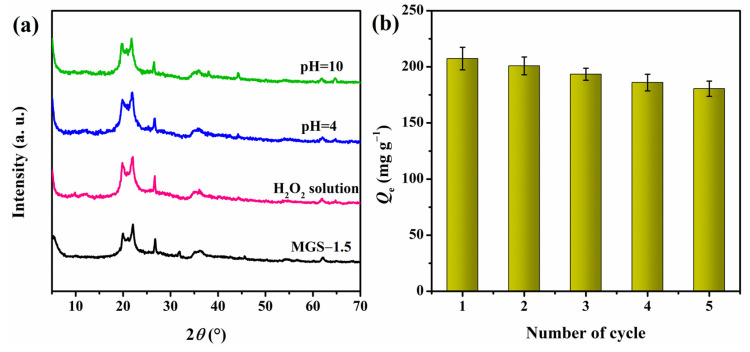
(**a**) XRD patterns of MGS-1.5 beads after adsorption in 100 mL 500 mg/L CR solution with various conditions, (**b**) adsorption capacity of regenerated MGS-1.5 beads at different cycle numbers.

Chemical stability is very important for the reusability of adsorbents. Herein, we evaluated the stability of the adsorbed beads by observing their SEM photos after adsorption at various conditions. First, CR solutions with pH = 4 and pH = 10 (adjusted by NaOH, strong corrosive) were used as the adsorption conditions, as shown in Figure 11a,b, the microstructures of MGS-1.5 beads changed little compared with the original structure (Figure 3), indicating the chemical stability of the MGS beads in the corrosive environment. In consideration of the oxidant environment, a CR solution with 10 mL H_2_O_2_ was used as the simulated wastewater, and the microstructure of MGS-1.5 beads after adsorption is shown in Figure 11c. It can be seen that even with a slight collapse appearing in the inner structure, the original porous structure and pore size were still retained, which are essential for the adsorption of dyes. In order to further determine the chemical stability of MGS-1.5 beads at various conditions, XRD patterns were recorded and the results are shown in Figure 12a. It was clear that there was no obvious change for MGS-1.5 beads after adsorption in CR solutions with various conditions, indicating the structural stability of the MGS-1.5 beads. Hence, regardless of corrosive or oxidative conditions, the MGS-1.5 beads can maintain good chemical stability.

Reusability is a vital factor for potential practical applications and can provide further insights into the adsorption mechanism. The adsorption–desorption process was performed five times, and the results are shown in Figure 12b. It is clear that the adsorption capacity for CR remained at approximately 87% of its initial adsorbability at the fifth cycle, indicating the benign and sustainable performance of MGS beads. Furthermore, the desorption using a strong base implies that the attachment of the CR molecules to the MGS beads was mainly through electrostatic interaction and ion exchange [54], which explains the pH-dependent adsorbability of the MGS beads.

## 4. Conclusions

This work reports the fabrication of cellulose/gelatin/sepiolite (MGS) gel beads via a simple and efficient two-step ‘hydrophilic assembly-floating droplet’ method. During the MGS gel bead preparation, microcrystalline cellulose (MCC) and gelatin (GEL) worked as dual network frameworks, and sepiolite (SEP) acted like a ‘crosslinker’ to connect and reinforce the dual network. A series of characterizations demonstrated that the MGS beads were lightweight and highly porous, and the incorporation of SEP, not only increased the adsorption capacity, but also made the beads more thermally stable. The adsorption behavior followed the pseudo-second-order model and Langmuir isotherm, with a maximum monolayer capacity of 279.3 mg·g^−1^ for CR at 303 K. Thermodynamic analyses illustrated that the CR adsorption onto MGS beads was spontaneous and endothermic. After five adsorption/desorption cycles, the MGS beads were found to retain 87% of their initial adsorbability, demonstrating themselves as an efficient and renewable candidate for dye wastewater treatment.

## Figures and Tables

**Table 1:** Kinetic parameters and experimental adsorption capacities for CR onto MGS-1.5 beads.

Kinetic Model		Pseudo-First-Order Model			Pseudo-Second-Order Model		
Initial Concentration (mg·L^−1^)	*Q*_exp_ (mg·g^−1^)	*Q*_1e_,cal (mg·g^−1^)	*k*_1_ (min^−1^)	*R* ^2^	*Q*_2e_,cal (mg·g^−1^)	*k*_2_ × 10^4^ (g·(mg min)^−1^)	*R* ^2^
100	69.2	69.4	0.0204	0.9501	77.6	3.0064	0.9934
300	164.5	174.9	0.0209	0.9394	187.6	1.0898	0.9905
500	205.6	202.8	0.0202	0.9560	229.9	1.0461	0.9940

**Table 2:** Langmuir and Freundlich parameters for CR adsorption onto MGS-1.5 beads.

Isotherm	Langmuir				Freundlich		
Temperature (K)	*Q*_max_ (mg·g^−1^)	*k*_L_ (L·mg^−1^)	*R* ^2^	*R*_L_	*k*_F_ (L·mg^−1^)	*n*	*R* ^2^
288	240.9	0.0050	0.9940	0.2500~0.8889	4.8914	1.7094	0.9751
303	279.3	0.0053	0.9938	0.2392~0.8830	6.1064	1.7304	0.9679
318	288.2	0.0054	0.9925	0.2358~0.8811	6.2682	1.7262	0.9657

**Table 3:** Thermodynamic parameters for CR adsorption at different initial concentrations.

Initial Concentration (mg·L^−1^)	Δ*H*° (kJ·mol^−1^)	Δ*S*° (J·(mol K)^−1^)	Δ*G*° (kJ·mol^−1^)		
			288	303	318
100	52.34	247.01	−18.80	−22.50	−26.21
300	13.90	102.81	−15.71	−17.25	−18.79
500	8.08	77.59	−14.27	−15.43	−16.59

## Data Availability

Data is contained within the article.
